# Swallowed secrets - Plastic bezoar-induced gastric outlet obstruction in a 14-year-old girl: A case report

**DOI:** 10.1016/j.ijscr.2024.110075

**Published:** 2024-07-24

**Authors:** Rajesh Kumar Parshuram Shrivastava, Abhishek Shrivastava

**Affiliations:** a Department of Surgery, Shreeji Hospital, Bhilad – Sanjan Road, Daheli-Bhilad, Taluka-Umargaon, Valsad, Gujarat 396105, India; b Nalanda Medical College and Hospital, Patna, Bihar, India

**Keywords:** Pica, Gastric outlet obstruction, Plastic bezoar, Pediatric surgery, Case report

## Abstract

**Introduction:**

Gastric outlet obstruction (GOO) is a rare but serious condition that can arise from various etiologies, including foreign body ingestion. We present a unique case of GOO in a 14-year-old girl resulting from the accumulation of plastic materials, known as a plastic bezoar, due to pica behavior.

**Case presentation:**

A 14-year-old girl with a history of pica presented with symptoms suggestive of acute gastric obstruction. Imaging studies revealed the presence of a large foreign body extending from the stomach to the jejunum, consistent with a plastic bezoar. Despite attempts at endoscopic removal, surgical intervention was ultimately required due to the size and location of the bezoar.

**Discussion:**

This case underscores the challenges associated with diagnosing and managing gastric outlet obstruction secondary to plastic bezoar formation, particularly in pediatric patients with underlying pica behavior. The diagnostic workup involved a multidisciplinary approach, including imaging studies and endoscopic evaluation. Surgical intervention, although invasive, proved necessary for definitive treatment in this case. Postoperative care focused on monitoring for complications and addressing the underlying pica behavior through psychological intervention and support.

**Conclusion:**

This case highlights the importance of early recognition, thorough diagnostic evaluation, and prompt intervention to prevent complications and ensure favorable outcomes. Collaborative efforts between medical and surgical teams are essential for the comprehensive management of such cases, emphasizing the need for tailored approaches to address both the physical and psychological aspects of care.

## Highlights

• Rare Case: A 14-year-old girl presented with gastric outlet obstruction caused by a large plastic bezoar, attributed to her history of pica.
• Presentation: Abdominal pain, vomiting, inability to tolerate oral intake, and a huge lump in the abdomen.
• Early detection and multidisciplinary care: Recognize issue early, undergo surgery, and ensure comprehensive patient care for best outcomes.
• Management: Surgical management is the mainstay, coupled to psychiatric evaluation and therapy to prevent recurrence.

## 1 Introduction

The term “bezoar” originates from the Arabic word “bazahr” or “badzehr,” which translates to antidote or counter-poison or an antidote [1]. Historically, animal bezoars were commonly used in medicinal practices until the 18th century. In humans, bezoars can consist of hair (trichobezoars), vegetable matter (phytobezoars), or a combination of both (trichophytobezoars) and are found in the gastrointestinal tract [2]. Although bezoars are rare in humans, they can cause significant complications such as gastrointestinal bleeding, intestinal obstruction, or perforation if not treated, with mortality rates potentially reaching 30 % [3]. Pica, the persistent eating of non-nutritive substances, is uncommon but causes significant disorder in children and adolescents [4].

This report describes a 14-year-old girl with a long-standing habit of ingesting plastic materials, resulting in a large plastic bezoar causing gastric outlet obstruction. She was managed in Shreeji hospital, a multifacility private hospital in Gujarat, India. This case underscores the importance of early diagnosis and intervention in preventing severe complications. The case has been reported in line with SCARE 2023 criteria [5].

## 2 Case report

A 14-year-old female presented with symptoms of abdominal pain, vomiting, and inability to tolerate oral intake. Her medical history, as given by her parents, was significant for pica, specifically the ingestion of plastic materials over several years. Physical examination revealed a distended abdomen with tenderness in the epigastric region. On palpation, a large hard lump was felt below the costal margin on the left side to the right hypochondrium.

### 2.1 Investigations

**Figs. 1 & 2:**
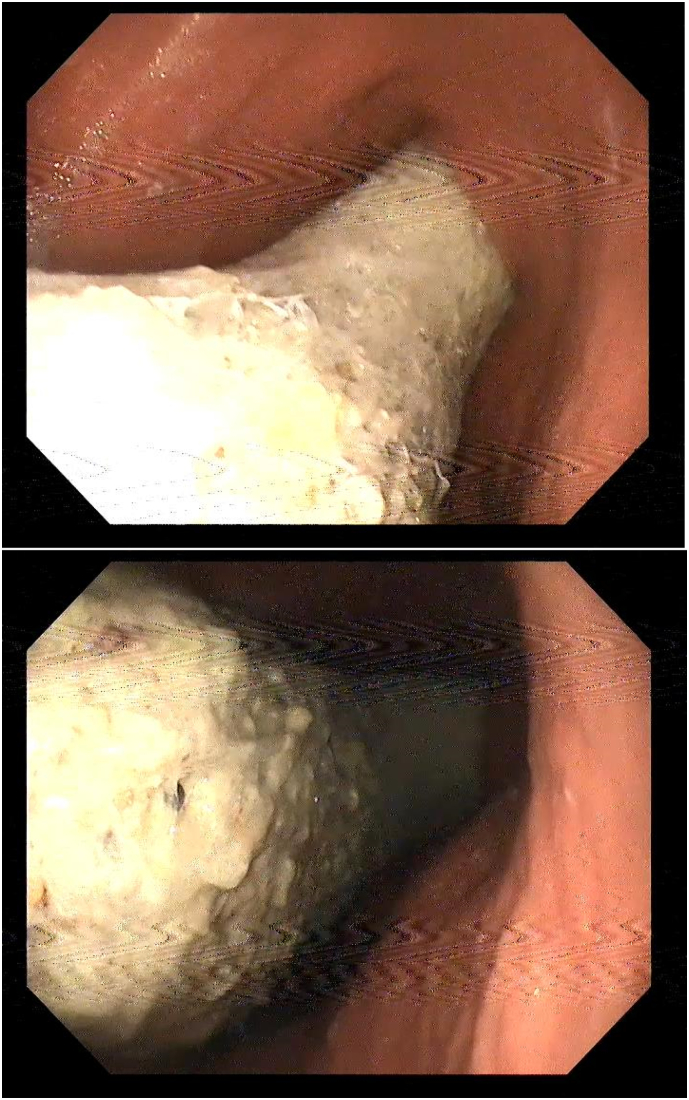
Endoscopic images.

**Figs. 3 & 4:**
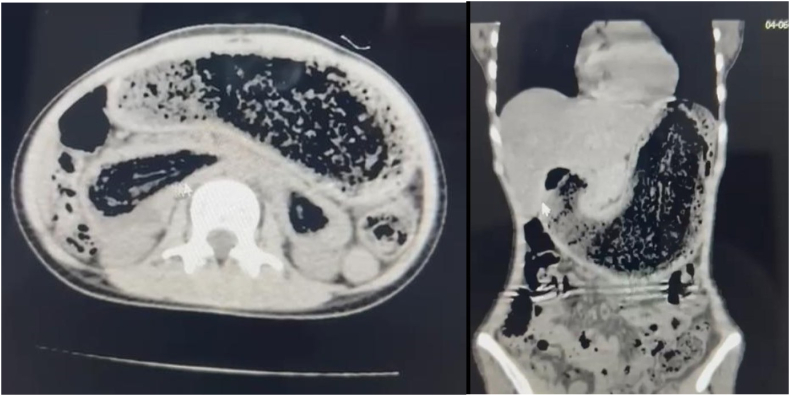
CT scan images.

Initial laboratory tests were unremarkable. A diagnostic endoscopy was performed due to the palpable lump in the epigastrium, revealing a large plastic foreign body completely filling the stomach and traveling through the pylorus (Figs. 1 & 2). A computed tomography (CT) scan of the abdomen confirmed a large plastic foreign body extending from the stomach into the jejunum, causing gastric outlet obstruction (Figs. 3 & 4).

### 2.2 Intervention

**Fig. 5:**
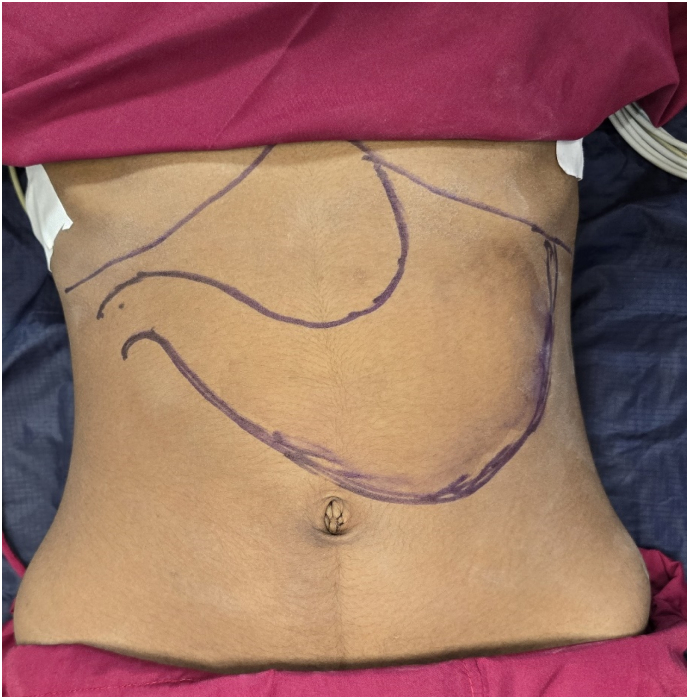
Surface marking of the lump.

**Fig. 6:**
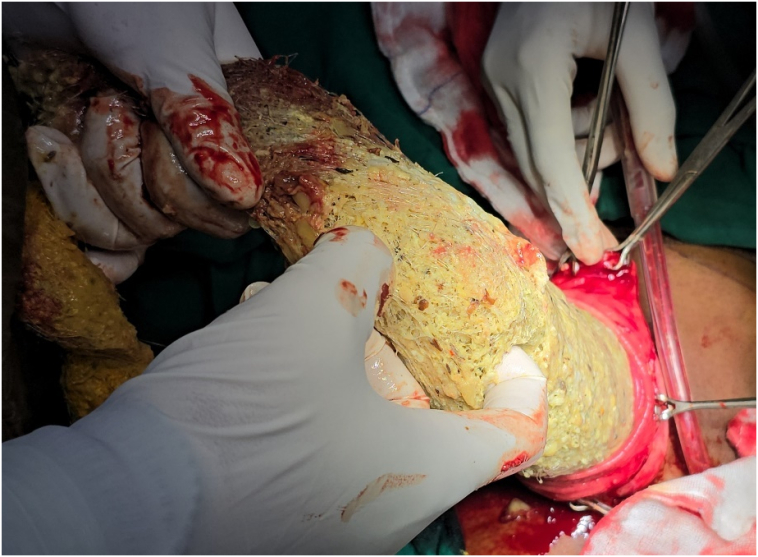
Removal of foreign body.

**Fig. 7:**
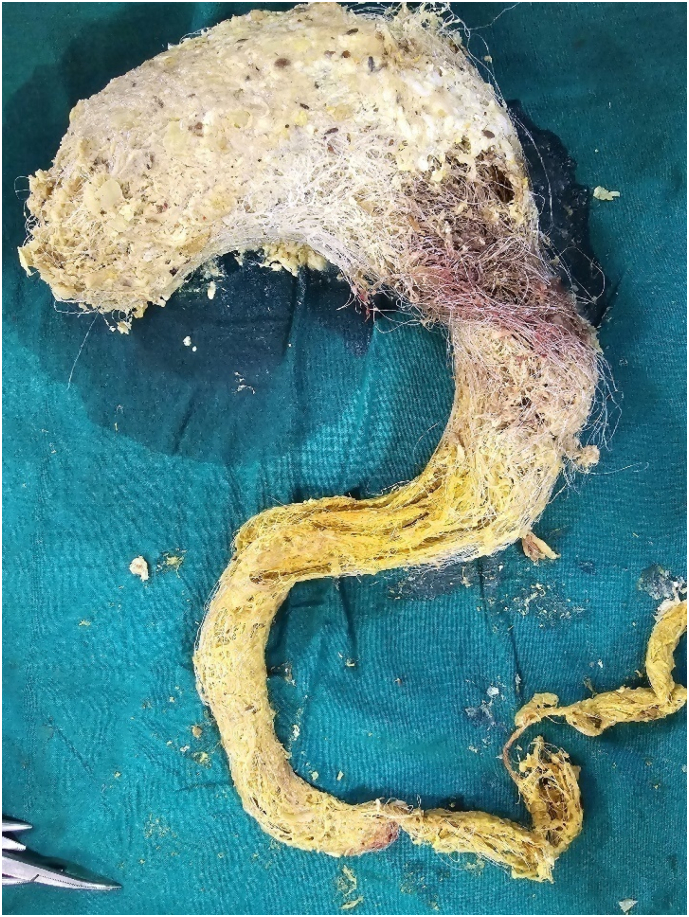
Removed foreign body.

**Fig. 8:**
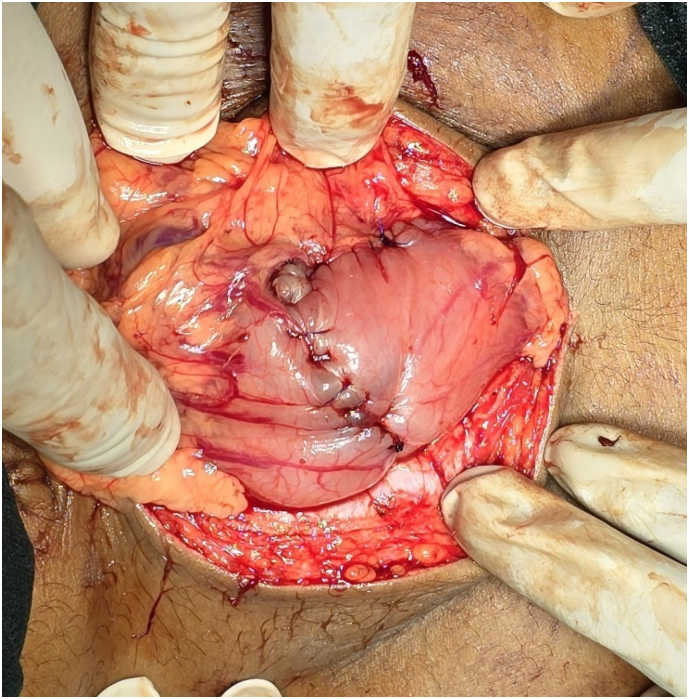
Closure of the stomach.

Given the size and extent of the bezoar (Fig. 5), endoscopic removal was not feasible. An open surgical approach was planned. Under general anesthesia, an upper midline incision was made, and a gastronomy was performed on the anterior wall of the stomach. A large, stomach/duodenum/jejunum-shaped plastic bezoar was identified, extending into the pyloric antrum and jejunum. The entire foreign body (Fig. 6) was carefully extracted in one piece (Fig. 7). Further endoscopic examination through the gastronomy confirmed that the jejunum was clear of any residual foreign material. The gastronomy was closed using polydioxanone (PDS) sutures in two layers (Fig. 8), and the abdomen was closed in layers. The presentation to surgery time was 3 days.

### 2.3 Outcome and follow-up

The patient had an uneventful recovery and was discharged on postoperative day four. At follow-up, she reported the resolution of her symptoms, and no recurrence of pica behavior was noted. She was referred to a psychiatrist for further evaluation and management of her pica.

## 3 Discussion

Bezoars, one of the rare benign causes of the gastric outlet obstruction [6], develops after ingestion of foreign material that accumulates in the gastrointestinal tract because of large particle size, indigestibility, gastric outlet obstruction, or intestinal stasis. The prevalence is less than 0.5 % for gastric bezoars. Most common types of bezoars known to cause gastric outlet obstruction are phytobezoars and trichobezoars. Other unusual bezoars are pharmacobezoars, lactobezoars, metal bezoars, plastic bezoars, and sand bezoars. Plastic bezoars, resulting from the ingestion of plastic material, has been described in our case report where the patient had swallowed a large amount of plastic material and since plastic cannot be digested, a bezoar formed in the stomach, and the long strings may extend down to the duodenum and intestines, leading to Rapunzel syndrome, as described by Vaughan et al. [7].

Predisposing factors to bezoars, in addition to dietary behavior, include previous gastric surgery, particularly partial gastrectomy or truncal vagotomy with pyloroplasty. In adults, bezoars are most frequently encountered after gastric operations. In children they are associated with pica, mental retardation, psychiatric disorder and coeliac disease [8,9].

Affected patients remain asymptomatic for many years. Symptoms develop as the bezoar increases in size. The most common presentations are abdominal pain, nausea/vomiting, obstruction and peritonitis. Less often, the patients present with weight loss, anorexia, hematemesis and intussusception. Complications by a large eroding or obstructing bezoar additionally include gastric ulceration, obstructive jaundice, acute pancreatitis and gastric emphysema [[10], [11], [12]].

The diagnosis often can be made on the basis of findings of conventional radiography and barium studies. On plain abdominal radiography, we found an opaque bezoar, which formed a perfect cast of the stomach. CT-scan could demonstrate a well-defined round mass which could be outlined by stomach or the bowel wall and present characteristic internal gas bubbles of bezoars [13].

The treatment of bezoars can be conservative, especially in the case of phytobezoars. Mechanical disintegration can be tried, using mechanical lithotripsy, a Dormia basket, or an electrosurgical knife [14]. Chemical dissolution is another option, with Coca-Cola® lavages or hydrolytic solutions [15]. Some authors have reported good results in the application of both methods [16]. Other operative treatments depend on the size, consistency, and location of the bezoar. Small bezoars can be removed with endoscopic treatment [17]. With larger bezoars, which can cause occlusion or bleeding, surgery is usually necessary. In these cases, laparoscopy or laparotomy is mandatory [18].

We have found limited case reports on plastic bezoars in literature. Sultan et al. [19] reported plastic foreign body causing bowel obstruction in a 58-year-old woman admitted for acute kidney injury, who underwent exploratory laparotomy and two points of impaction were noted: the first was in the small bowel and the second point in the stomach. Verma VK [20] described a 14 year male with upper abdomen swelling where X-ray of the abdomen showed the features of intestinal obstruction and sonography revealed a huge homogeneous lump. An exploratory laparotomy was undertaken and two separate lumps of wires were found in the stomach and the jejunum. Yaka M et al. [21] reported a case of a 14 year old girl with abdominal computed tomography (CT) scan showing a heterogeneous gastric mass and at laparotomy, a bezoar made up of plastic material extended throughout the length of the stomach and small bowel with two perforations of jejunum. The bezoars was removed encase through gastronomy with assisted multiple enterotomies.

## 4 Conclusion

This case report illuminates the critical challenges in managing gastrointestinal bezoars, particularly those induced by plastic ingestion, underscoring the necessity for a comprehensive approach. In this case, the patient's pica behavior directly led to a serious medical complication. Integration of psychiatric evaluation and addressing underlying behavioural issues are crucial in preventing recurrence and optimizing long-term management.

Strengths in the management include prompt recognition of symptoms, thorough diagnostic workup involving imaging and endoscopy, and timely surgical intervention when endoscopic removal is not feasible. However, the absence of standardized protocols for such cases and potential limitations in accessing specialized surgical expertise present notable weaknesses.

## Consent

Written informed consent was obtained from the patient for publication of this case report and accompanying images. A copy of the written consent is available for review by the Editor-in-Chief of this journal on request.

## Parental consent

Written informed consent was obtained from the patient's parents/legal guardian for publication and any accompanying images. A copy of the written consent is available for review by the Editor-in-Chief of this journal on request.

## Ethical approval

Not applicable. Our institution does not require ethical approval for reporting individual cases or case series.

## Funding

No sponsors are involved in this study.

## Guarantor

Dr Rekha Kumari Rajesh Shrivastava.

## CRediT authorship contribution statement

Primary author - Dr Rajesh Kumar P Shrivastava is the treating & operating surgeon in the above case report, has collected the data and worked on the study concept.
Dr Abhishek Shrivastava - Corresponding author.

## Declaration of competing interest

The authors declare no conflict of interest.
